# Barriers to women’s disclosure of domestic violence in health services in Palestine: qualitative interview-based study

**DOI:** 10.1186/s12889-020-09907-8

**Published:** 2020-11-26

**Authors:** Amira Shaheen, Suzy Ashkar, Abdulsalam Alkaiyat, Loraine Bacchus, Manuela Colombini, Gene Feder, Maggie Evans

**Affiliations:** 1 Division of Public Health, Faculty of Medicine and Health Sciences, An Najah National University, Nablus, Palestine; 2 Faculty of Public Health & Policy, Department of Global Health and Development, London School of Hygiene & Tropical Medicine, 15-17 Tavistock Place, London, WC1H 9SH UK; 3 Centre for Academic Primary Care, Population Health Sciences, Bristol Medical School, Faculty of Health Sciences, Canynge Hall, 39 Whatley Road, Bristol, BS8 2PS UK

**Keywords:** Domestic violence, barriers to help-seeking, health professionals’ training, disclosure of violence, qualitative study, Palestinian women

## Abstract

**Background:**

Domestic violence (DV) damages health and requires a global public health response and engagement of clinical services. Recent surveys show that 27% of married Palestinian women experienced some form of violence from their husbands over a 12 months' period, but only 5% had sought formal help, and rarely from health services. Across the globe, barriers to disclosure of DV have been recorded, including self-blame, fear of the consequences and lack of knowledge of services. This is the first qualitative study to address barriers to disclosure within health services for Palestinian women.

**Methods:**

In-depth interviews were carried out with 20 women who had experienced DV. They were recruited from a non-governmental organisation offering social and legal support. Interviews were recorded, transcribed and translated into English and the data were analysed thematically.

**Results:**

Women encountered barriers at individual, health care service and societal levels. Lack of knowledge of available services, concern about the health care primary focus on physical issues, lack of privacy in health consultations, lack of trust in confidentiality, fear of being labelled ‘mentally ill’ and losing access to their children were all highlighted. Women wished for health professionals to take the initiative in enquiring about DV. Wider issues concerned women’s social and economic dependency on their husbands which led to fears about transgressing social and cultural norms by speaking out. Women feared being blamed and ostracised by family members and others, or experiencing an escalation of violence.

**Conclusions:**

Palestinian women’s agency to be proactive in help-seeking for DV is clearly limited. Our findings can inform training of health professionals in Palestine to address these barriers, to increase awareness of the link between DV and many common presentations such as depression, to ask sensitively about DV in private, reassure women about confidentiality, and increase awareness among women of the role that health services can play in DV.

**Supplementary Information:**

The online version contains supplementary material available at 10.1186/s12889-020-09907-8.

## Background

Domestic violence (DV) damages health, requiring a public health response and engagement of clinical services. It is estimated that 3% of women globally have experienced physical and/or sexual violence from a partner during their lifetime [1]. This prevalence is raised to 37% in Eastern Mediterranean countries [1, 2]. A recent systematic review showed that a doubling of this lifetime prevalence (70%) was reported among Arab women attending health care settings in Arab countries [3].

Looking specifically at Palestine, a 2019 survey by the Palestinian Centre Bureau of Statistics (PCBS) found that 27% of currently married or ever married Palestinian women had at least one experience of some form of violence from their husbands in the 12 months preceding the interview [4]. Psychological violence was the highest at 52%, economic 41%, social 33%, physical 17%, and sexual 7%. Sixty-one per cent of interviewed survivors had not disclosed violence either formally or informally. 24% of survivors took refuge in their parents’ or siblings’ homes and a further 20% did not leave their homes, but asked for help from either their parent or relative [4]. Six per cent sought advice from a work colleague or a neighbour. Despite 40% of interviewed survivors reporting that they were aware of the existence of support services, only 5% had sought formal help, generally from the police or legal services [4].

The above figures make no reference to help-seeking via health services, despite the serious health consequences of DV such as depression, sleep problems, abortion, pain, and hypertension [4–9]. DV results in substantial social and economic costs related to treating the physical and psychological impacts on women, absence from work, reduced quality of life, and problems with integrating into society [10, 11]. Eliminating DV by 2030 is the second item of the UN 5th Sustainable Development Goals (SDGs), which would make a major contribution to women’s health [12].

Health services could potentially play an important role in supporting women who are experiencing violence [13]. This can be done within the clinical setting and through referral of identified women to specialist services [14]. However, studies in the UK, India and Malaysia reveal that women experience barriers to help-seeking and disclosure of violence in the clinical setting. Barriers include self-blame, shame and embarrassment, prior negative experiences of help-seeking, fear of the consequences of disclosure, economic dependency on the perpetrator and lack of awareness about formal support services related to DV [15–17]. Similar barriers were identified among women from eastern Mediterranean countries [18].

Studies show that women who have experienced DV are more likely to disclose to a health care provider if asked in an empathic, non-judgemental way [19]. Little is known about how Palestinian women view the role of health professionals in responding to DV, or how they feel about disclosing their experience of violence in health care settings. The study reported here aimed to articulate Palestinian survivors’ of DV attitudes towards and experiences of disclosure in a health setting. It is the first qualitative study to explore barriers to disclosure of DV among Palestinian women survivors of violence.

## Methods

As a part of a larger mixed-method study aimed at enhancing the Palestinian primary health care response to DV, we conducted 20 semi-structured interviews with Palestinian women survivors of violence. This article presents results from the qualitative interviews.

### Recruitment and sample

The Women’s Centre for Legal Aid and Counselling (WCLAC) in the Occupied Territories of the West Bank of Palestine was contacted to help with recruitment of women survivors of DV. WCLAC provides legal and social support services, free of charge, to survivors of DV. Purposive sampling was used in order to recruit as wide a range of women as possible. Eligible women were aged 18 and over, and were receiving support from WCLAC following exposure to DV. To maximise geographical and social diversity, two centres were used for recruitment, one located in Ramallah and the other in Hebron. Two social workers in each location, aiming at a range of age and marital status, approached survivors and asked for their consent to be interviewed. Women who agreed to participate were interviewed by AS or Rania Abu-Aaita, a member of the research team who received training in qualitative interviewing.

### Interview procedure

After written informed consent, women were interviewed by one of two female researchers in a private room provided by the NGO. All women were given transport costs to attend the interview. Semi-structured interviews, using a piloted topic guide, were conducted to explore women’s personal experiences with DV, influences on their decisions to disclose violence, and their experience of talking to health care providers (HCPs) about violence and abuse. The Topic Guide was developed specifically for this study. Further information about the topics covered in the interview is given in the Topic Guide: [see Additional file 1]. Interviews were conducted in Arabic and lasted for 1 to 2 h. A social worker was available on site for any women in need of support during or after the interview.

### Qualitative analysis

Interviews were audio-recorded and were translated and transcribed verbatim into English. This enabled members of the international multi-lingual research team to advise, quality check and provide mentoring in the skills needed for qualitative analysis. A sample of the transcribed interviews was checked against the Arabic recording, by the first author, to ensure accuracy. Data from transcripts were anonymised and analysed using reflexive thematic analysis, following the method developed by Clarke and Braun [20, 21]. This is an interpretive approach whereby meaning is generated through the researchers’ interpretation of the data, rather than using a priori expectations [22]. It also takes account of the context and influence each researcher brings to their interpretation. AS and SA were Palestinian residents, whose direct experience of cultural influences on women might have influenced their interpretation. An inductive approach was taken to the analysis, using the constant comparison technique [23]. Transcripts were read and re-read by three members of the team, ME, SA and AS, who noted emergent topics and themes. These were discussed and a coding frame was developed as a group process. The coding frame was amended as new transcripts were analysed. A sample of transcripts were double coded for verification. NVIVO 11 was used to facilitate the coding and analyses.

Trustworthiness and rigour of the study were maintained by ensuring credibility, dependability and transferability [24]. The credibility of this study was accomplished via peer checking and a prolonged engagement in the data over six months. Dependability was achieved by an audit trail. Transferability was accomplished by selecting participants who had direct experience of the phenomena under investigation [25].

## Results

### Characteristics of participants

All the contacted women agreed to participate. Most of the women were in marriages arranged by their families and the majority were separated or divorced. Although three women self-defined as ‘Married’ (ie: Still in the relationship) rather than ‘Separated’ or ‘Divorced’, none of them were living with their husband at the time of interview. Thirteen women had children living at home with them (between one and seven children per household), one was pregnant at the time of interview, and four women had children who were no longer at home. The husband was identified as the main perpetrator by all the interviewed women. Three women also identified in-laws as secondary perpetrators. Further details are given in Table 1.

**Table 1:** Socio-demographic characteristics of the study participants (*N* = 20)

Characteristics	***n***
**Age (years)**	
20–29	8
30–39	8
40–49	3
50–59	1
**Educational status**	
Primary	3
Secondary	7
University	5
Vocational education	5
**Employment status**	
Currently employed	3
Unemployed	17
**Marital status (self-defined)**	
Separated^*^	10
Divorced	7
Married	3
**Type of marriage**	
Traditional^**^	18
Modern	2
**Length of marriage**	
Less than 10 years	12
More than 10 years	8

^*^Separated: not officially divorced, ^**^Traditional: family arrangement

### Barriers to survivors disclosing DV (DV) to health care providers

Eleven out of the 20 interviewed survivors said that they had disclosed DV to HCPs at some point. However, all the women encountered multiple barriers to talking to HCPs about their experiences that either prevented disclosure or made it difficult. Themes were identified by the authors that reflect the cultural barriers to talking about DV that pervade all areas of women’s lives and experiences.

### Survivors’ individual level barriers

Two key individual barriers that were identified were women’s sense of dependence on their husband and their fear of the consequences of disclosure.

### Dependence on their husband

Women described their dependence on their husbands, both financially and in terms of cultural expectations about how a married woman should behave. They were reluctant to talk about relationship difficulties, matters considered ‘too personal’, and likely to result in them being blamed, shamed or embarrassed. None of the women were living with their husbands at the time of interview. Although some were already divorced, others were contemplating whether or not to take this step.

*“I felt like I would be blamed for it [relationship difficulty with her husband] and people might say; look, she let out secrets between her and husband. Why would she say that? So that would make me shy, embarrassed to talk about it” [woman aged 20–29, Hebron].*

*“Because I don’t want to expose the secrets of my home and I want to stay protected (under husband) and live in comfort” [woman aged 20–29, Hebron].*

Feeling ‘protected’ by marriage was also financial. Exposing violence in the home might lead to separation and divorce, with no guarantee of financial support for the woman or her children. Fear of living apart from their children was a major barrier for women to disclosure.

*“Because my family, if I divorced, don’t want me to keep his children and I don’t want to lose my daughters. If I lose the children I will suffer” [woman aged 30–39, Hebron].*

The data highlighted the cultural taboo about women speaking out against their husbands and the stigma attached to those who do so, especially if they are separated or divorced. Most women found it hard to talk about DV to family members or friends, as well as to HCPs, and they often hid their injuries, or their cause, from them, as well as from HCPs. One woman went to the hospital with cuts on her legs, and she hid the reason for her injuries.

*“They asked me what happened. I didn’t say it was done by my husband, I said it happened while I was cutting a piece of wood” [woman aged 30–39, Hebron].*

Staying in the abusive relationship and keeping quiet about the violence can be seen as active, calculated choices to protect themselves and their children, expressing agency in difficult circumstances, until an opportunity arose to take action.

### Fear

Women talked about their fear of escalation of the violence should they disclose.

*“because the violence would get worse and the problems would increase. The problems between my family and his would become worse and God forbid, if it got to the point where they might hurt each other, someone might get killed … that’s not a little issue.” [woman aged 40–49, Ramallah].*

Fear of what might happen if they left the house by themselves to seek help or if they talked about the violence, kept women at home, unable to access HCPs or other forms of help.

*“Researcher: you didn’t tell the doctor that you couldn’t go out? SW20: no. Researcher: why? SW20: honestly, I was very, very scared of him … Researcher: you were scared of him? SW20: yes. My husband used to put his key in the door, and when I’d hear it, when I’d hear the key enter the door, I’d start shaking” [woman aged 30–39, Ramallah].*

Women were also reluctant to seek help, especially for psychological distress, for fear of being labelled ‘mentally ill’. Losing their credibility as a competent wife or mother might lead to them losing custody of their children.

*“Researcher: so you would like to see a psychologist but you’re worried your husband will find out and … SW14: he’d say, for example, ‘she’s mentally sick!’ He won’t say, ‘she wants to change her behaviour’, he’d say ‘this woman is mentally sick. I want to take the kids’.” [woman aged 30–39, Ramallah].*

*“Other than that, family, or society rather considers psychiatrists are only for crazy people. That’s their negative views on psychological treatment” [woman aged 20–29, Ramallah].*

### Health care service level barriers

#### Expectations of health care providers’ (HCP) role

Women varied in their views as to whether attending to the issue of DV was within the remit of HCPs, and they had low expectations of getting help. Women saw the focus on physical health as the normative role of HCPs, and they were unwilling to take up HCPs’ time in busy clinics.

*“I don’t know. The idea never came to my mind. I don’t expect that they could help me with the situation” [woman aged 20–29, Ramallah].*

*“yes I had the potential to [disclose], but I didn’t feel like the doctor would listen to me if I did … because there were a lot of people waiting for a turn and other than that, she works fast … she doesn’t ask about the person’s state … it was just an exam for the fetus, and that’s it, work is done” [woman aged 20–29, Ramallah].*

Some women expressed personal ambivalence about the idea of HCPs probing into areas of their life they considered ‘private’, even when they had obvious bruising. One woman was relieved that the doctor did a ‘normal’ physical examination, asking no questions about her bruising.

*“it was normal, anyway, he checked me out, put his stethoscope here. Examined my arms and said alright up, we are done … .. because maybe I want a doctor just to examine me, not to know everything about me” [woman aged 30–39, Hebron].*

*“honestly? I don’t like them to get involved in my private life...I consider it my personal life. It’s private” [woman aged 30–39, Ramallah].*

Others, however, said they would prefer HCPs to look beyond their physical health, show concern for women’s psychological well-being and take the initiative to ask about DV. These women wished that, when they were in the hospital with signs of DV, the HCPs would ask them about how they were feeling and give them a chance to talk about DV.

*“maybe they could ask me questions, give me support …*” *[woman aged 20–29, Hebron].*

*“Well, women feel that they don’t care. I don’t feel like they care about these things at all. So at least they should ask those that he feels something might be wrong.” [woman aged 20–29, Hebron].*

Most women were clear that the initiative must come from the HCP asking direct questions, even repeatedly, in order to overcome their initial reluctance to disclose.

*“Researcher: in a case where a woman refused to talk about, what should the doctor do? SW03: ask the first time, and second time, third time” [woman aged 30–39, Hebron].*

#### Women’s direct experiences of disclosure and non-disclosure to HCPs

Missed opportunities for disclosure were described by women who presented with warning signs of abuse including low mood, bruising and poor nourishment, with no questions asked by HCPs.

*“There was an apparent thing on my arm, it was obvious that I had. When I get upset it’s obvious, he didn’t ask me about it or anything” [woman aged 30–39, Hebron].*

These women wished that, when they were in the hospital with signs of DV, the HCPs would ask them about how they were feeling and give them a chance to talk about DV.

One woman’s obvious distress was ignored. Despite her tears, the HCPs carried on ‘as normal’.

*“I was crying but, its normal, no one asked me about it. About anything!” [woman aged 20–29, Hebron].*

However, in spite of the barriers, just over half of these survivors had disclosed violence to HCPs.

*“no, she asked me. she asked me “what’s going on? I feel like you’re not all right?” I told her “I’m having some problems with my husband.” [woman aged 30–39, Ramallah].*

Many of them, however, reported little benefit. Simply initiating a conversation was felt to be insufficient, some women felt that HCPs should make a full assessment of the violence.

*“What happened, why it happened. They should do a proper assessment about anything that looks like a case of abuse. It’s obvious when something is normal and something is strange.” [woman aged 30–39, Hebron].*

Women often said they wanted help to change their husband’s behavior, so that they could preserve their marriage, and their social and financial survival. They did not know who to call on other than involving the police and filing a complaint.

*“SW15: yes. If I knew that there was someone that could talk to my husband and influence him to change his temper habits and such, then I would say something, but there is no one” [woman aged 20–29, Ramallah].*

They wanted HCPs to take responsibility for making a report of DV to the police, according to an unwritten and rarely followed mandate from the Ministry of Health.

*“[HCPs should] do what they’re supposed to. For example, I reach out to them and they suspect that something is happen, they should immediately call the police. Even if I’m insistent upon not wanting to talk, from their report and their examinations, it’s clear” [woman aged 20–29, Ramallah].*

*“They said the results show that nothing is broken there is only fracture. The Doctor asked me: “how this happened”, and I said that it was from my husband”. The Doctor then asked for the police. The police arrived and they asked me everything.” [woman aged 30–39, Hebron].*

#### Privacy, confidentiality and trust

Lack of personal privacy in health care consultations was another barrier to disclosure for the women, who were often accompanied by their husband or in-laws, making it impossible for them to talk about DV.

*“At the hospital they asked me what was wrong. My mother in law told me “if you tell them ‘he hit me’ we’ll divorce you. Don’t say that. We don’t have women who complain about their husbands here.” The doctor told me “I know you’ve been hit. I know it, but if you don’t want to say something, I can’t do anything.” I went back home …” [woman aged 20–29, Ramallah].*

*“Researcher: did you have privacy when the doctor came to examine you? Were you by yourself with him, for example? I mean, could you have told him “honestly, doctor, I … SW11: No my mother in law was with me …*. *there’s no privacy pause and even if I told him, the doctor how could he benefit me? He won’t help me with anything” [woman aged 20–29, Hebron].*

Women did not trust patient confidentiality, and were anxious about disclosing in case ‘DV’ appeared in writing in their medical report. They described overlapping social and professional networks in their communities. HCPs may know other family members, social ties and loyalties might outweigh concepts of confidentiality, and disclosure may not remain a private matter. For women, this risked an escalation of violence and other repercussions.

*“SW10: yes I was very scared. Researcher: why, what’s he going to do to you? SW10: what do you mean? if he finds out that I told the doctor, I’m sure he wouldn’t stay quiet. I mean, [the doctor] is going to say something. He’s going to say “this girl gets hit while pregnant” and [word of] that gets to him [my husband], then he [husband] would have hit me even more if he found out that I talked to the doctor about him.” [woman aged 20–29, Hebron].*

*“For example, if my medical record was with you, and your colleagues came along she’ll read the file and know about my life” [woman aged 30–39, Hebron].*

*“I didn’t want to make any problem. The Doctor is our family doctor and I was ashamed to talk about this” [woman aged 30–39, Hebron].*

*“No. I wouldn’t have trusted him because I’m living with people I don’t trust; he might be one of them. I mean, he’ll always side with them; he is still from their town. I wouldn’t have trusted him or told him. I’d be scared to go tell my husband, then he’d tell me why did you go tell him and the situation would be flipped against me.” [woman aged 20-29n, Hebron].*

### Societal level barriers to disclosure

#### Normalization of violence

Women described the cultural expectation that a wife should tolerate her husbands’ behavior, that it is ‘normal’ to be hit by him. An abusive husband should be given a ‘chance to change’.

*“Yes, we are afraid of the society, we always give a chance that maybe things will change. Unfortunately, it is the opposite, it will carry on and on until it becomes serious.” [woman aged 30–39, Hebron].*

*“Listen, a divorced woman is always the one blamed. No matter what. They don’t say that the man was no good, no it’s the woman fault. You should have been patient. You should have tolerated it. What am I supposing to tolerate more than I have endured?” [woman aged 20–29, Hebron].*

#### Expectations of women’s role

Women described their society as ‘repressive’ and they were fearful of being judged if they talked about DV or left their husbands.

*“Honestly no, because I never thought I’d ever file a complaint against him in my life. Firstly, for the sake of my kids and then because women are always violated. No matter what you say, your name and subject is going to be on the tip of everyone’s tongues” [woman aged 30–36, Hebron].*

One woman whose husband was in prison for DV felt she was being watched by the community and always had to be on her best behavior, as if she were the guilty party. Others echoed this experience of being abandoned by society for speaking out. Fear of being blamed and being seen as a ‘home-wrecker’ stopped some women from filing for divorce.

*“I think it’s more the nature of our society. Our society abandons a woman who speaks out about her circumstances, even when they are bad” [woman aged 30–39, Hebron].*

This woman felt wrongly blamed for speaking up about DV from her husband, who she addresses as ‘you’.

*“Like I said before they put all the blame on the woman and it’s because of you, like how they’ve already put all the blame on me. The closest people to you; you’re the reason, you’re the home wrecker” [woman aged 30–39, Hebron].*

One woman regretted not having spoken out sooner, since she now recognized how her rights had been taken away since marriage.

*“no, now I would have talked. I would have talked then because I’ve given up so many things in my life, from the day I married [perpetrator] until now, there are a lot of rights that he’s denied me from” [woman aged 30–39, Hebron].*

#### Stigma

Women’s fear of being stigmatized for their actions was a strong theme in their accounts. They described their fear of the stigma of a ‘mental health label’ or of being a ‘home-wrecker’, and of being ostracized by society for speaking out against their husband, separating from or divorcing him. After leaving a violent relationship, women continued to feel stigmatized and faced barriers to getting support for themselves or their children, such as attending counselling sessions alone or getting psychological help for themselves or their children.

*“Even for my son and his sessions, in the beginning I told her even if you need to put two sessions a week, do it. I wanted my son to get better, but I felt that it [son receiving counselling] was unaccepted” [woman aged 40–49, Hebron].*

## Discussion

Barriers to women survivors’ help-seeking for DV in the Occupied Palestinian Territories involve a complex interplay of factors at several levels (individual, service and societal). Studies in other countries have led to the development of a multi-level conceptual framework for understanding help-seeking among survivors of DV [15, 26]. This framework suggested by Liang provided a model for guiding the data analysis in this study. A significant finding is that normative cultural values about women’s role in Palestinian society exert a strong influence, leading to barriers to disclosure of DV at all levels of women’s experience, the individual, service and societal.

Although the majority of the women in the study had left their abusive relationship, their pathways to support had been through legal channels rather than health care. Having found freedom, some women regretted not having challenged cultural norms sooner and taken opportunities to disclose, for example when presenting to HCPs with injuries caused by DV. However, women’s agency to be proactive in help-seeking or trying to change their situation, is clearly limited. Women felt disempowered in their marriages, and the only answer was for their husband’s behaviour to somehow change. Meanwhile, their own actions were often tactical, motivated to ensure damage limitation for them and their children.

The context of women’s lives was the largely patriarchal and hierarchical structure of the Palestinian family. Customs and behaviours ensure that men maintained their dominant social roles over women, a concept for which Connell coined the phrase ‘hegemonic masculinity’ [27]. Men expect their wives and children to respect them, and to comply with their roles and demands. In this context there are many examples of the normalisation of DV, and few opportunities are available for women to talk about DV either in their informal networks or to professionals [28, 29]. These difficulties were compounded by overlapping social and professional networks and the custom of family members accompanying women to healthcare appointments, leading to a lack of trust in disclosure to HCPs and a lack of the privacy to do so. Family members, HCPs and other members of the community might all subscribe to a ‘conspiracy of silence’ around such uncomfortable issues. Fear of making matters worse, being subjected to even more abuse, of being ostracised by society and losing their children were all significant barriers to women speaking out about abuse.

Women described feelings of embarrassment and shame from disclosure, reflecting that DV is perceived as a private issue, and that sharing experiences with others would not be accepted [30, 31]. Lack of awareness of possible help that can be obtained from health care services was a further constraint on survivor disclosure. This is perhaps not surprising given the findings of a companion study interviewing HCPs and health officials in Palestine, carried out alongside the current study. This revealed the lack of clear DV guidelines for HCPs, and protocols for how to respond to disclosure, which were recognized as challenges to the health service’s response to DV [32]. However, all the women who were interviewed had found a way to get help from a professional agency, although the role of HCPs was limited. The determination of the women to break free meant that the majority were living apart from their perpetrator at the time of interview. In spite of the barriers highlighted in this study, a systematic review [3] suggests that Arab women still view visiting the health care setting as socially acceptable, and once trust is gained, and confidentiality is granted between them and their health care provider, disclosure will follow.

Women’s experience of domestic violence in the Occupied Palestinian Territories and difficulty in obtaining support, is exacerbated by the violence created by the existence of the occupying settler state [33]. Women are particularly vulnerable to violence from male perpetrators who are themselves living in a situation of conflict and violence, leading to a sense of disempowerment [34]. Women also suffer geographical suppression whereby access to health care facilities is difficult if they have been displaced from their usual territory or have to cross checkpoints [35].

In order to decrease DV, a focus group of young Palestinians from Gaza recommended raising awareness among Palestinian women toward their legal rights and the available services [36]. Awareness raising must, however, go alongside measures to address societal norms towards women and gender roles. Other studies also indicate the importance of awareness campaigns in introducing available services to women victims of violence and their communities in an attempt to raise the level of help seeking [28, 29].

This Palestinian study adds to the findings of previous qualitative studies that highlight the importance of sensitivity in the timing and questioning about DV by HCPs, who must be alert to windows of opportunity. Previous studies stress the importance of a woman’s personal ‘readiness’ to disclose DV, at a time that is right for her [37, 38]. The health system itself must also be ‘ready’ to take on responsibility for helping women survivors of DV, with suitable infrastructure, training and referral pathways. A recent study in Lower and Middle Income countries (LMIC) explores the concept of health systems ‘readiness’ and corroborates many of the findings in the present study as regards health service barriers to disclosure [32].

The role of health care systems in responding to DV and in facilitating access to support services has been demonstrated worldwide [19]. Our findings can inform training of HCPs in Palestine to facilitate asking about DV and responding appropriately.

### Strengths and limitations

#### Strengths

This is the first study to investigate barriers to disclosure among Palestinian women in the Occupied Territories of the West Bank who are exposed to DV.

The use of thematic analysis that starts with coding, grouping of codes under specified themes, investigating and defining these themes by more than one researcher, gives an in-depth view of the survivor experience that reflects on their collective experience rather than an individual one.

### Limitations

Data for this study were collected by interviewing women in two legal centres, who had successfully sought help, hence the results might not reflect all the barriers that are experienced by women.

Women who visited these legal centres may have been more severely affected by DV.

As the recruitment of participants were at voluntary basis, it is possible that those who agreed to be interviewed were the more comfortable with the topic being investigated, or that those who refused to participate were more severely affected and scared.

## Conclusions

Training of Palestinians HCPs on response to DV should be tailored to address the barriers to disclosure experienced by survivors, for example, how to ask sensitively about DV in private, the importance of reassuring women about confidentiality, and increasing awareness of the link between DV and many common presentations such as depression. Actions such as securing private spaces in clinics, for women to feel safe to disclose, and increasing awareness among women of the role that health services can play in DV is crucial.

## Supplementary Information

**Additional file 1:** doc: Topic Guide for Interviews with Women Survivors.

## Data Availability

The data that support the findings of this study are available from the corresponding author upon reasonable request.

## Abbreviations

DV: Domestic Violence
GBV: Gender Based Violence
HCP: Health Care Professional
PCBS: Palestinian Central Bureau of Statistics
SDG: Sustainable Development Goals
WCLAC: Women’s Centre for Legal Aid and Counselling
NGO: Non-Governmental Organisation
LMIC: Lower and Middle Income Countries
