# Supplementary material for: Barriers to women’s disclosure of domestic violence in health services in Palestine: qualitative interview-based study
Source: BMC Public Health. 2020 Nov 26;20:1795. doi: 10.1186/s12889-020-09907-8 (PMC7691108; doi:10.1186/s12889-020-09907-8)
Supplement: Supplementary file 1 — Additional file 1: doc: Topic Guide for Interviews with Women Survivors. [file 12889_2020_9907_MOESM1_ESM.docx]

Additional File 1: Topic guide for interviews with women survivors

**Preparation and Safety Issues**

- Ensure that the interview takes place in a confidential environment where the conversation cannot be overhead.
- Introduce yourself, which organisation you are from and what the study is about *(‘exploring women’s views to understand how primary health care professionals can best support women who are experiencing violence and abuse’*).
- An explanation of why her name was put forward for an interview and where from (i.e. that she has received support from WCLAC)
- Give the woman the Participant Information Sheet to read and ask her whether she has any questions. If she is happy to proceed, ask her to sign the consent form. If a woman is worried about signing her name, let her know that she can mark an ‘X’ or write her initials to indicate that she understands what the study involves and is happy to participate.
- It may not be safe for the woman to take the Participant Information Sheet away with her. **Check this with her**. If it is not safe, you can suggest that she saves the Principal Investigator’s name (Amira or use a code name) and office number (on the PIS) in her mobile phone, just in case she has any questions later on. Again, check that it is safe for her to store the number.
- Reassure the woman about issues of confidentiality and anonymity. Let her know that if she uses the real names of people and organisations, that the research team will remove these names during transcription.
- Thank her for agreeing to take part.
- Before starting the interview, you may want to say the following: *“I want you to feel relaxed and comfortable during the interview. I am interesting in learning from your experiences of seeking help. Although I have a set of questions, please feel free to tell me anything that you think is important. If anything is unclear during the interview, please stop me and I’ll explain. If any of the questions make you feel uncomfortable, just tell me and we’ll move to another question. You don’t have to answer anything that you are not comfortable with”.*

Date of interview:

Unique identification number: WOM

Consent obtained: Yes No

Place where interview took place:

Interviewer name:

***Before we start the interview, I’d like to ask some questions that help to describe you***

1. **Age 5. Anyone else living at home with you Yes**
2. **Civil status**

Married No

Engaged

**5b. If yes, who**

Divorced Parents Others (specify)

Widowed Siblings
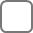


Single In-laws

**2a. If married, for how long?**

Years/Months

**2b. If married, was it 6. Highest level of education**

Traditional Primary school

Modern Secondary school

1. **Live with husband** University

Yes Professional qualification
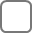


No Other (specify)

1. **Children 7. Currently in paid work**

Yes Yes

No No

**4a. If yes, how many children** **7a. What do you do**

**7b. How long have you been doing this**

**4b. If yes, how many live with you**

**7c. How many hours do you work a week**

***Thank you. Now I’m going to ask you about your personal experiences. Please let me know if any questions make you feel uncomfortable or you prefer not to answer.***

1. **Experiences of violence and abuse**
2. I am aware that you have received support from WCLAC because someone close has hurt or frightened you. Can you tell me who this person is?

**Probes:**

- Is there anyone else at home that has hurt or frightened you?

1. Do you feel comfortable telling me what happened?

**Probes for behaviours if needed:**

- Can you tell me about some of the behaviours and actions of [X] that frighten you or make you feel unsafe at home?
- Has [X] stopped you from doing things like visiting your family and friends, having a job, pursuing further education?
- Has [X] called you hurtful names, shouted, screamed at you, done something to humiliate you?
- Has [X] physically hurt you? For example, hitting, pushing, slapping, kicking, pulling your hair?
- Has your husband ever physically forced you to have sexual relations? [If yes] Do you feel comfortable telling me about it? [**Researcher:** If woman shows signs of distress at this question, do not probe further – “*it’s okay you don’t have to answer, we can move on*”]

1. Roughly how long have you been experiencing these behaviours from [X]?
2. Do you have access to your own money?

**Probes:**

- Do you need to ask permission to use this money? **[If yes]** from who?

***Thank you for sharing that, as I understand that it can be difficult to talk about. How do you feel? Is it okay for me to continue? Okay, the next questions are about talking to primary health care professionals about the violence and abuse.***

1. **Talking to primary health care providers**
2. Who do you normally see at the clinic when you are ill or have a health appointment? For example, a doctor, a nurse, midwife, others? [**Researcher**: It’s fine if it’s more than one]
3. Did you ever talk to any of these health care professionals about the violence and abuse?**(IF NO, NEVER TALKED TO HEALTH CARE PROFESSIONALS GO TO Q11)**
4. **IF YES, she talked to a health care professional about violence and abuse**
5. Which health care professional(s) did you talk to?
6. How did that conversation come up?

**Probes:**

- Did you tell them or did they ask you directly about violence and abuse at home?
- Talk me through what you remember about that conversation

1. What made you decide to talk to the [doctor/nurse/midwife/other] at this particular time?

**Probes:**

- Was there something happening in the relationship? Can you tell me about that?
- Have you experienced any health issues that might be related to the violence and abuse (e.g. feeling tired, feeling depressed, headaches, physical injuries). **[If yes]** Can you tell me about that?

1. What did you hope for when you told the [doctor/nurse/midwife/other]?
2. How did they respond to you?

**Probes:**

- What did they do or say?
- Did they offer any information or referrals to any organisations?**[If yes]** What did they offer?
- Is there any help or support you would have liked that the health professional did not offer? **[If yes]** Can you tell me about that?
- What did you like or dislike about the way they responded?
- Did you feel understood, comforted?
- What happened after? Did you follow-up on the information or referrals offered **[If yes]** what was the outcome of that?

**[RESEARCHER]: If the woman talked to more than one health care professional, try to explore each event separately without causing burden to her, using the probes above. Ask her if she found one health professional more helpful than another and explore the reasons why. You could start with: *“You said you also talked to a nurse, can you tell me about that?”***

1. Are you taking any prescription medications for health problems that might be related to the violence and abuse? For example, pain killers, anti-depressants, sleeping tablets.

**Probes if yes:**

- How long have you been taking them?
- Do they help you at all? [If yes] In what way?

1. **IF NO, she never talked to a health care professional about abuse**
2. What makes it difficult for you to talk to a health care professional aboutyour experiences?

**Probes:**

- Do you have enough privacy when you visit the clinic? Is there usually someone accompanying you? Can you speak to a health care professional alone?
- Did you feel there is enough time during your appointments to talk about it?
- Would you prefer to talk to a female or male health care professional?
- Are you worried about other people finding out that you have told someone? **[If yes]** What do you think would happen?
- Do you have trust and confidence in any of the health care professionals you see? Tell me more about that.

1. Were there any times when you tried to talk to a health care professional about the violence and abuse, but you felt that they were not listening? **[If yes]** Can you tell me about that?
2. Would you like your health care professional to ask you if someone at home is hurting you?

**Probes:**

- Do you feel that you could raise the issue yourself, or is it important that they ask you?
- How does it make you feel that your health care professional does not ask you whether someone at home is hurting you?

1. Have you experienced any health issues that might be related to the violence and abuse (e.g. feeling tired, feeling depressed, headaches, physical injuries). **[If yes]** Can you tell me about that?
2. Are you taking any prescription medications for health problems that might be related to the abuse? For example, pain killers, anti-depressants, sleeping tablets.

**Probes if yes:**

- How long have you been taking them?
- Do they help you at all? [If yes] In what way?

1. **Views on the role of primary health care professionals**

***We are interested in women’s views about what primary health care professionals should be doing to support women have had similar experiences to you.***

1. What do you think primary health care professionals (like doctors, nurses, midwives etc...) should do to support women who are experiencing violence and abuse?

**Probes:**

- What should their role be?
- Examples: listening, referring to other organisations, talking to the person who is being violent or abusive?

1. How do you feel about primary health care professionals (like doctors, nurses, midwives) asking women about violence and abuse at home?

**Probes:**

- When do you think is the best moment for primary health care professionals to ask these questions?
- If a woman chooses not to tell the health care professional, should they ask again?
- Do you think primary health care professionals should ask *all women* about violence and abuse at home? Or should they ask just, some women who they suspect? Why do you think this?

1. Would you want your health care professional to record your experiences of violence and abuse confidentially in your medical record?

**Probes:**

- Do you see any benefits of recording it?
- Do you think there are any risks?

1. **Referrals to other organisations**

***Just to re-cap, you spoke to A, B, C and was referred to D[(researcher adapt as needed]***

1. Can you tell me about any other organisations that you have contacted to get support or protection? (e.g. legal, social worker, shelters, etc)

**Probes if yes contacted other organisation(s):**

- How did you find out about them?
- What did you hope for when you contacted them?
- Did they offer you any choices? **[If yes]** Can you tell me about them?
- What did you like or dislike about the way they responded? Did you feel understood, comforted, or not?
- Did you feel or do anything differently as a result of contacting those organisations? **[If yes]** Can you tell me about that?
- Is there any help or support you would have liked that these organisations did not offer? **[If yes]** Can you tell me about that?
- How did the referral process work? Did you have to wait long to be seen? Do you think the process needs to be improved or is it okay as it is?

1. **Feelings about the present**
2. How are things for you now? Has anything changed for you?
3. What would you like to happen in the future?
4. Of all the people and organisations you told about your experiences, who did you have the most confidence and trust in? Can you explain why?
5. **Closing the interview sensitively**

***Thank you. We are coming to the end of the interview now. Before we end I’d like to know:***

1. How do you feel about our conversation today? What feelings has it brought up?
2. Would you find it helpful to talk to someone from WCLAC]?I can arrange this for you.
3. If it’s safe, we can send you a short summary of the findings. Are you interested in this? **[Researcher: if yes, find out how this can be done safely]**

***Thank you for sharing your experiences and helping with our research. I know that it can be very difficult and painful to talk about. It takes a lot of strength to be open with someone that you do not know and I really appreciate it. Is there anything else you would like to tell me before we end the interview?***
